# Testosterone Usage Leading to Pulmonary Embolisms and Deep Vein Thrombosis: A Case Report and Review of the Literature

**DOI:** 10.3390/hematolrep15020029

**Published:** 2023-04-26

**Authors:** Sasmith R. Menakuru, Mona Atta, Vijaypal S. Dhillon, Ahmed Salih

**Affiliations:** Department of Internal Medicine, Indiana University School of Medicine, Muncie, IN 47306, USA

**Keywords:** pulmonary embolism, testosterone, deep vein thrombosis, androgens

## Abstract

Androgen usage has widely increased in recent times via prescribed and unprescribed means. Testosterone is a popular androgen taken by both athletes and the general population. While there is some evidence of androgens being thrombogenic, we report on a 19-year-old male who presented to the hospital after the usage of testosterone for one month, leading to the development of multiple pulmonary emboli and deep vein thrombosis. The authors hope to elucidate the relationship between testosterone usage and thrombosis formation.

## 1. Introduction

Androgens are utilized by bodybuilders and nonathletes alike to increase muscle mass, improve performance, and enhance physical appearance. Androgen use has spread from the realm of competitive sports to everyday individuals wanting to gain a competitive edge. Unfortunately, this practice has not been without consequences. The usage of androgenic steroids poses a serious global health concern in current times. In a meta-analysis of 187 studies, the prevalence of usage was 3.3% and was higher in males (6.4%) compared to females (1.6%) [1]. The study also revealed that usage was higher among recreational athletes than professional athletes (18.4% compared to 13.3% respectively) [1]. In the United States, the average age of androgen usage is in the early 20s, and it is estimated that at least one million men have experienced dependence on androgens [2]. Studies by Buckman et al. and Ip et al. found that individuals who were dependent on androgens were more likely to have coexisting substance abuse and mental health disorders [3,4].

The clinical impact of androgen therapy is multifold, beginning with the unsafe means of accessing steroids. People who use androgens may obtain products from the Internet or via illegal means. The unsafe means of access include obtaining steroids via laboratories, prescribed steroids meant for other individuals, and obtaining those intended for veterinary purposes. The method of steroid administration varies among users. Athletes who use the drugs will typically use them in two different patterns known as pyramiding (taking the steroid in escalating doses) and/or stacking (combining two or more steroids) [5]. Some users may take additional medications to counteract the side effects of steroid use. These include human growth hormones used to counteract a reduction in testicular size, aromatase inhibitors to prevent gynecomastia, and 5-alpha reductase inhibitors to prevent balding [6].

Multiple types of androgens are utilized, including testosterone esters (enanthate and cypionate, taken by injection), 17-alpha-alkylated androgens (known as androgen-anabolic steroids (AAS), such as oral stanozolol or parenteral nandrolone), and androgen precursors (androstenedione and dehydroepiandrosterone). The most commonly utilized androgens are testosterone, trenbolone, and a veterinary steroid called boldenone [6]. Additionally, selective androgen receptor modulators are nonsteroidal drugs developed to increase binding at androgen receptors in certain tissues, such as muscles. These compounds have not been approved by any country; however, they are widely available on the Internet.

Pulmonary embolism (PE) is a seemingly unrelated condition that occurs at a rate of over ten million cases per year, with significant morbidity and mortality if left untreated. PE has a mortality rate of more than 15% in the first 3 months following diagnosis [7]. The incidence of PE is higher in males compared to females and much higher in the elderly above the age of 75 [8]. The common presenting symptoms of PE are tachycardia, dyspnea, calf pain due to associated deep vein thrombosis (DVT), syncope, pleuritic chest pain, and cough with hemoptysis [9]. Wells criteria and the pulmonary embolism rule-out criteria (PERC) are tools utilized to determine the pretest probability of patients with PEs. D-dimer elevation can also point towards the possibility of PE or DVT because it is a breakdown product of blood clotting. If the pretest probability is high, the evaluation of a PE should be performed by utilizing computed tomography pulmonary angiography or a ventilation-perfusion scan, which is preferred in patients with elevated creatinine. Patients with a high pretest probability should be provided supplemental oxygen and stabilized. After a diagnosis is made via imaging, anticoagulation should be started. Heparin is the treatment of choice in a hospital setting. Patients can be discharged on warfarin or direct oral anticoagulants such as apixaban. In patients with life-threatening PEs, reperfusion therapy via thrombolysis or embolectomy is performed with consideration of the patient’s bleeding risk [10]. In patients with unprovoked PE or those with increased risk factors, anticoagulation should be continued indefinitely [10].

There have been several case reports in which patients who were taking androgenic steroids developed pulmonary embolisms, but the relationship is still poorly defined [11,12,13]. The typical risk factors for PE and DVTs are advanced age, malignancy, pregnancy, venous stasis, obesity, and heritable hypercoagulation disorders. The typical adverse effects of androgens have been well documented, including liver toxicity, polycythemia, cardiac dysfunction, psychiatric symptoms, acne, testicular atrophy, and gynecomastia [2]. Here, the authors report a case of a 19-year-old male who had been taking intramuscular testosterone for one month, which was obtained via illicit means to gain muscle mass. The patient presented complaints of shortness of breath, tachycardia, and calf pain to the hospital and was subsequently found to have multiple pulmonary emboli and a deep vein thrombosis. The authors hope that this case informs clinicians and athletes about the possible side effects of exogenous androgen use.

## 2. Case Report

A 19-year-old male with no past medical history presented complaints of shortness of breath, fatigue, and severe calf pain to the emergency department. He had a muscular build and said that he was a mixed martial arts fighter who was training diligently for an upcoming tournament. He denied the use of any medications, either prescribed or over the counter. He denied any travel history, trauma, recent surgeries, prolonged immobilization, sick contacts, or any significant family history. Routine laboratory investigations were performed, and an immediate CTPA was taken. An electrocardiogram was also performed, which revealed sinus tachycardia. 

**Figure 1:**
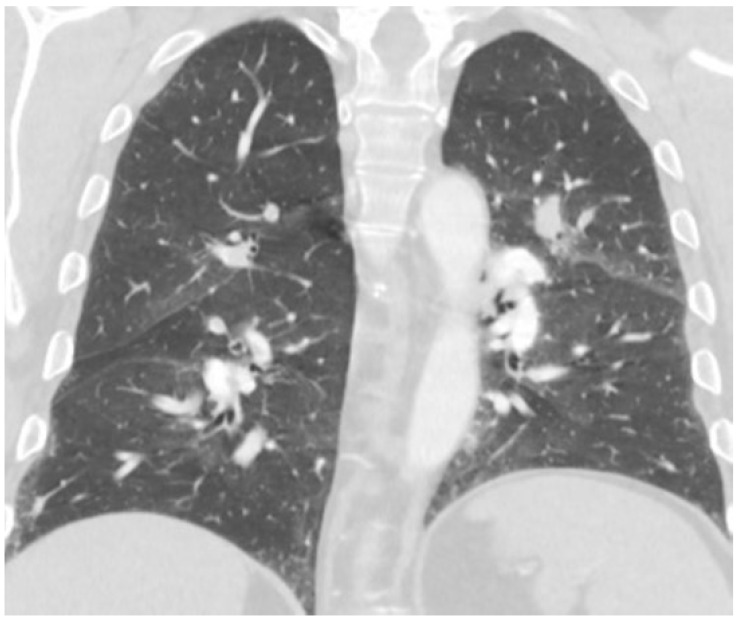
Left lung pulmonary embolism.

**Figure 2:**
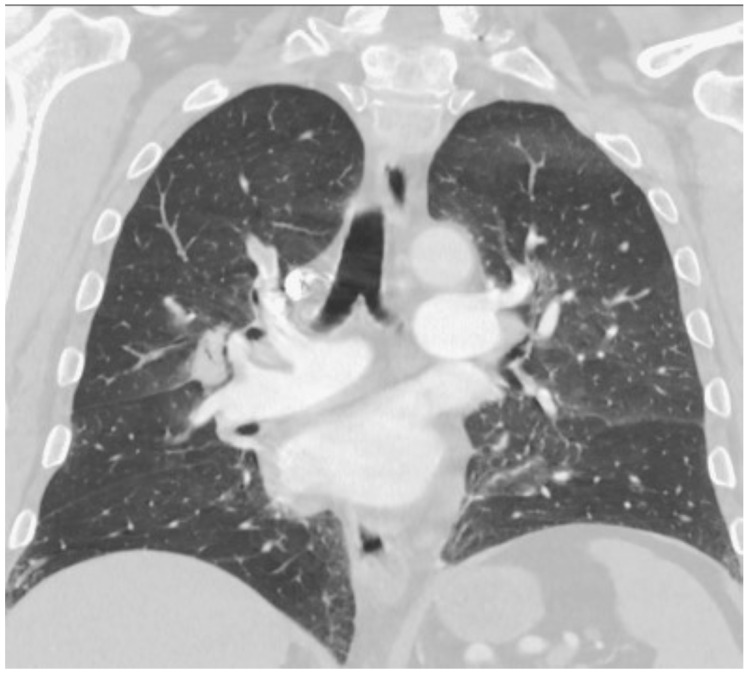
Right lung pulmonary embolism.

The CT demonstrated extensive segmental and subsegmental pulmonary emboli throughout all lobes of the lungs. There were also findings of pulmonary arterial hypertension and right heart strain. Bilateral upper lobe perihilar nodular consolidations with surrounding ground glass attenuation favoring pulmonary infarcts were observed (Figure 1 and Figure 2). A Doppler of the patient’s left lower extremity revealed deep vein thrombosis (DVT) in the peroneal vein. Given his findings of multiple PEs and a DVT, he was started on a continuous heparin infusion.

Routine investigations revealed a hemoglobin of 21.4 g/d (13.8–17.2), a white blood cell count of 7.0 × 109/L (4.5–11.0), and platelets of 279 × 109/L (150–400). He had a D-dimer of 3235 ng/mL (<500), ferritin of 494 ng/mL (24–336), and erythropoietin of 117.4 mIU/mL (4–26), but all other values were within normal limits. His prothrombin time (PT) was 12.0 s (11–13.5), his international normalized ratio (INR) was 1.07 (<1.1), and his activated partial thromboplastin time (aPTT) was 30.4 s (21–35). Results were negative for factor V Leiden, antiphospholipid antibodies, antinuclear antibodies, protein C, protein S, and factor VIII, and he did not have an antithrombin deficiency. Janus kinase 2 (JAK2) gene testing to rule out polycythemia vera was also negative. Due to his young age, with no significant past medical history, no oncological risk factors, and no smoking history, the overall risk of malignancy was determined to be low. In addition, the patient did not have any underlying cardiopulmonary or renal disease.

Upon examination, the patient appeared muscular in build; however, he had gynecomastia. Apart from tachycardia, other exam findings were benign, including non-enlarged lymph nodes. When the patient was asked if he was taking any supplements, he was initially adamant in denying the use of performance-enhancing drugs. However, after admission, he asked to speak with his physician team again, at which point he admitted to using intramuscular testosterone that he had acquired illicitly while abroad.

The patient was monitored with telemetry, and hematology was consulted. Heparin infusion continued for two more days while he was an inpatient in the progressive care unit. The patient was counseled about the harmful effects of androgens, including the possibility of causing thrombosis. He was then discharged on apixaban 10 mg twice a day for seven days and then 5 mg twice a day thereafter for at least three months. He reported for follow-up with hematology after three months, with a repeat CTPA that showed resolution of the prior pulmonary embolisms. His hemoglobin had returned to normal limits. The patient was able to stop taking the apixaban at that time, as his pulmonary embolisms were likely provoked by his androgen use. 

## 3. Discussion

Testosterone and other androgen usage have increased in the 21st century due to widespread use ranging from athletes to adolescents with the aim of increasing athleticism and muscularity [14,15]. As this practice continues to grow in popularity, clinicians should be aware of the potential side effects of steroid use. In current clinical practice, androgens are utilized in males with primary hypogonadism, hypogonadotropic hypogonadism, androgen deficiency, and postpubertal cryptorchidism. In females, androgens can be utilized in the treatment of breast cancer, endometriosis, postpartum breast pain, and fibrocystic breast disease. In both males and females, androgens can be utilized in hereditary angioedema, bone pain due to osteoporosis, human immunodeficiency virus wasting syndrome, muscular dystrophy, and to promote weight gain after illness. 

The use of testosterone and other androgens has been found to cause polycythemia due to the stimulation of erythropoiesis, as observed in the current case. Erythrocytosis and polycythemia can be used interchangeably, and they are defined by an increase in the absolute red blood cell mass in the body [16]. Polycythemia leads to increased blood viscosity, which can have negative outcomes such as increased thromboembolic risk and major adverse cardiac events. However, there is less evidence of the adverse effects of polycythemia due to testosterone therapy [17]. Studies have shown that polycythemia may also have systemic effects on venous return and cardiac function [18]. Testosterone-induced polycythemia must be differentiated from polycythemia vera, which can be performed by first checking an EPO level. If the EPO level is high, then secondary causes of polycythemia should be looked for; however, if levels are low, JAK2 mutation testing should be performed. If JAK2 is positive, then polycythemia vera can be considered, but if it is negative, a bone marrow aspirate and biopsy can be carried out.

There are multiple proposed hypotheses as to how polycythemia occurs with the use of testosterone. Initially, it was thought to be caused solely by the stimulation of erythropoietin (EPO) production by the kidneys. However, several recent studies have disproved this, including one by Maggio et al., which showed that people who took testosterone had an increase in hemoglobin without a significant elevation of EPO [19]. Therefore, there is growing evidence that erythrocytosis is due to a multifactorial process. Bachman et al. suspected that the mechanism of erythrocytosis was due to the suppression of hepcidin and ferritin, leading to increased iron absorption, transport, and subsequent erythropoiesis [20]. Bachman et al. further researched this and proposed that testosterone stimulates EPO transiently and suppresses hepcidin, creating a new EPO set point at a higher level than one set at the physiologic hemoglobin [20]. The authors have concluded that the red cell mass was increased by testosterone via the inhibition of BMP-Smad signaling in hepatocytes, leading to the suppression of hepcidin. Testosterone stimulates the renal secretion of EPO, stimulating erythropoiesis, which then further suppresses hepcidin. This, in turn, causes a cascade of upregulation of GATA-1 (erythroid transcription factor) and GATA-dependent genes, which may increase EPO sensitivity and stimulate stress erythropoiesis [21]. Estradiol, a breakdown product of testosterone via aromatase, is also thought to be a cause of polycythemia. A study conducted by Calado et al. found that estradiol increased hematopoietic telomerase [22]. In patients taking testosterone, its breakdown would lead to increased estradiol and increased telomerase, leading to increased hematopoietic proliferation [23].

A study carried out by Ory et al. showed that men who were on testosterone therapy and developed secondary polycythemia had a higher risk of major cardiac adverse events and venous thromboembolism during the first year of therapy [24]. A literature review performed by Jones Jr. et al. revealed that men who were undergoing testosterone replacement therapy had a 315% increased risk of the development of erythrocytosis as compared to the control group, but the investigation of its association with VTE was inconclusive [23]. In a review conducted by Ohlander et al., there was evidence that short-acting injectable formulations of testosterone led to the highest incidence of erythrocytosis, and the associated high blood viscosity increased the potential for vascular complications [25]. In a study of transgender men using testosterone by Madsen et al., it was found that erythrocytosis was common, with a particularly increased occurrence in individuals who used tobacco [26]. There was also an increase associated in transgender men with an increased body mass index, and in those who injected testosterone [26]. Another study conducted by Martinez et al. with a case–control study of 928,745 patients showed a significantly increased incidence rate of venous thromboembolism within the first six months of testosterone therapy in men without a prior history of thromboembolic risk [27].

The other mechanism by which testosterone and other androgens could possibly lead to thrombogenesis is through platelet activity; however, current data on this is sparse. In a study conducted by Ajayi et al., there was a demonstration of testosterone-increasing thromboxane A2 (TxA2), which acts through membrane surface receptors to aggregate platelets and constrict vascular smooth muscle in both in vitro and in vivo studies [28]. Ajayi et al. found that the peak level of TXA2 was noted four weeks after testosterone treatment and would return to baseline in the eighth week; this increase in platelet density was statistically significant [28]. A study conducted by Banerjee et al., comparing the effects of testosterone on healthy male and female volunteers, concluded an increase in ADP-2, decreased platelet nitric oxide, and increased platelet TxA2 synthesis only in male volunteers [29]. Exogenous androgens lead to platelet activation and aggregation via the enhancement of platelet cyclooxygenase activity, an increase in platelet TxA2 receptor density and synthesis, an increase in platelet-activating factor, and a decrease in platelet nitric oxide level [30].

Polycythemia in patients utilizing testosterone therapy may be observed with increases in hematocrit after one month of therapy and further increases in a dose-dependent fashion [31]. The side effects of polycythemia are due to hyperviscosity and include blurry vision, paresthesia, fatigue, and headaches, the most significant of which is thrombosis [32]. Studies by Basaria et al. [33], Finkle et al. [34], and Vigen et al. [35] all found there was a higher risk of major cardiac adverse events with testosterone, which prompted the FDA to issue a label change to reflect increased risks of myocardial infarction and stroke for patients taking testosterone. Considering the aforementioned studies, the DVT and multiple PEs occurring in the current case were likely multifactorial—as a result of decreased hepcidin, the stimulation of platelets through TXA2, increased estradiol, and increased EPO—leading to polycythemia and consequent thrombosis. Currently, there is no data to support the need for prophylactic anticoagulation in patients with secondary erythrocytosis due to testosterone. In patients presenting with polycythemia secondary to the use of testosterone, reducing the dose of testosterone or utilizing phlebotomy may be implemented. Patients with an essential need for testosterone and thrombosis would require anticoagulation. The authors of this case hope that there will be further research on the effects of testosterone therapy on thrombosis, given the increase in usage via both prescribed and illicit means. In patients with unexplained thrombosis, the possibility of using testosterone or other androgens cannot be excluded.

## Data Availability

Not applicable.

## References

[1] Sagoe D., Molde H., Andreassen C.S., Torsheim T., Pallesen S. (2014). The global epidemiology of anabolic-androgenic steroid use: A meta-analysis and meta-regression analysis. Ann. Epidemiol..

[2] Pope H.G., Wood R.I., Rogol A., Nyberg F., Bowers L., Bhasin S. (2014). Adverse health consequences of performance-enhancing drugs: An Endocrine Society scientific statement. Endocr. Rev..

[3] Buckman J.F., Farris S.G., Yusko D.A. (2013). A national study of substance use behaviors among NCAA male athletes who use banned performance enhancing substances. Drug Alcohol Depend..

[4] Ip E.J., Lu D.H., Barnett M.J., Tenerowicz M.J., Vo J.C., Perry P.J. (2012). Psychological and physical impact of anabolic-androgenic steroid dependence. Pharmacotherapy.

[5] NIDA How Are Anabolic Steroids Used? 22 November 2021. https://nida.nih.gov/publications/research-reports/steroids-other-appearance-performance-enhancing-drugs-apeds/how-are-anabolic-steroids-used.

[6] de Ronde W., Smit D.L. (2020). Anabolic androgenic steroid abuse in young males. Endocr. Connect..

[7] Turetz M., Sideris A.T., Friedman O.A., Triphathi N., Horowitz J.M. (2018). Epidemiology, Pathophysiology, and Natural History of Pulmonary Embolism. Semin. Interv. Radiol..

[8] Horlander K.T., Mannino D.M., Leeper K.V. (2003). Pulmonary Embolism Mortality in the United States, 1979–1998: An analysis using multiple-cause mortality data. Arch. Intern. Med..

[9] Stein P.D., Beemath A., Matta F., Weg J.G., Yusen R.D., Hales C.A., Hull R.D., Leeper K.V., Sostman H.D., Tapson V.F. (2007). Clinical Characteristics of Patients with Acute Pulmonary Embolism: Data from PIOPED II. Am. J. Med..

[10] Di Nisio M., van Es N., Büller H.R. (2016). Deep vein thrombosis and pulmonary embolism. Lancet.

[11] Lee K., Toraby S., Cotterman R., Oriowo B., Fish J. (2019). A Tumultuous Course of Exogenous Testosterone by a Bodybuilder Causing a Catastrophic Hypercoagulable State in the Surgical Intensive Care Unit. Case Rep. Vasc. Med..

[12] Liljeqvist S., Helldén A., Bergman U., Söderberg M. (2008). Pulmonary embolism associated with the use of anabolic steroids. Eur. J. Intern. Med..

[13] Nguyen S.M., Ko N.K., Sattar A.S., Ipek E.G., Ali S. (2017). Pulmonary Embolism Secondary to Testosterone-Enhancing Herbal Supplement Use. Cureus.

[14] Handelsman D.J. (2021). Androgen Misuse and Abuse. Endocr. Rev..

[15] Montisci M., El Mazloum R., Cecchetto G., Terranova C., Ferrara S.D., Thiene G., Basso C. (2012). Anabolic androgenic steroids abuse and cardiac death in athletes: Morphological and toxicological findings in four fatal cases. Forensic Sci. Int..

[16] Pillai A.A., Fazal S., Babiker H.M. (2022). Polycythemia. [Updated 10 July 2022]. StatPearls.

[17] McMullin M.F., Bareford D., Campbell P., Green A.R., Harrison C., Hunt B., Oscier D., Polkey M.I., Reilly J.T., Rosenthal E. (2005). Guidelines for the diagnosis, investigation and management of polycythaemia/erythrocytosis. Br. J. Haematol..

[18] Wells R.E., Merrill E.W. (1962). Influence of Flow Properties of Blood Upon Viscosity-Hematocrit Relationships. J. Clin. Investig..

[19] Maggio M., Snyder P.J., Ceda G.P., Milaneschi Y., Luci M., Cattabiani C., Masoni S., Vignali A., Volpi R., Lauretani F. (2013). Is the haematopoietic effect of testosterone mediated by erythropoietin? The results of a clinical trial in older men. Andrology.

[20] Bachman E., Feng R., Travison T., Li M., Olbina G., Ostland V., Ulloor J., Zhang A., Basaria S., Ganz T. (2010). Testosterone suppresses hepcidin in men: A potential mechanism for testosterone-induced erythrocytosis. J. Clin. Endocrinol. Metab..

[21] Bachman E., Travison T.G., Basaria S., Davda M.N., Guo W., Li M., Westfall J.C., Bae H., Gordeuk V., Bhasin S. (2014). Testosterone Induces Erythrocytosis via Increased Erythropoietin and Suppressed Hepcidin: Evidence for a New Erythropoietin/Hemoglobin Set Point. J. Gerontol. Ser. A Biomed. Sci. Med. Sci..

[22] Calado R.T., Yewdell W.T., Wilkerson K.L., Regal J.A., Kajigaya S., Stratakis C.A., Young N.S. (2009). Sex hormones, acting on the TERT gene, increase telomerase activity in human primary hematopoietic cells. Blood.

[23] Jones S.D., Dukovac T., Sangkum P., Yafi F.A., Hellstrom W.J. (2015). Erythrocytosis and Polycythemia Secondary to Testosterone Replacement Therapy in the Aging Male. Sex Med. Rev..

[24] Ory J., Nackeeran S., Balaji N.C., Hare J.M., Ramasamy R. (2022). Secondary Polycythemia in Men Receiving Testosterone Therapy Increases Risk of Major Adverse Cardiovascular Events and Venous Thromboembolism in the First Year of Therapy. J. Urol..

[25] Ohlander S.J., Varghese B., Pastuszak A.W. (2018). Erythrocytosis Following Testosterone Therapy. Sex. Med. Rev..

[26] Madsen M.C., van Dijk D., Wiepjes C.M., Conemans E.B., Thijs A., Heijer M.D. (2021). Erythrocytosis in a Large Cohort of Trans Men Using Testosterone: A Long-Term Follow-Up Study on Prevalence, Determinants, and Exposure Years. J. Clin. Endocrinol. Metab..

[27] Martinez C., Suissa S., Rietbrock S., Katholing A., Freedman B., Cohen A.T., Handelsman D.J. (2016). Testosterone treatment and risk of venous thromboembolism: Population based case-control study. BMJ.

[28] Ajayi A.A.L., Mathur R., Halushka P.V. (1995). Testosterone Increases Human Platelet Thromboxane A _2_ Receptor Density and Aggregation Responses. Circulation.

[29] Banerjee D., Mazumder S., Bhattacharya S., Sinha A.K. (2014). The sex specific effects of extraneous testosterone on ADP induced platelet aggregation in platelet-rich plasma from male and female subjects. Int. J. Lab. Hematol..

[30] Roşca A.E., Vlădăreanu A.M., Mititelu A., Popescu B.O., Badiu C., Căruntu C., Voiculescu S.E., Onisâi M., Gologan Ş., Mirica R. (2021). Effects of Exogenous Androgens on Platelet Activity and Their Thrombogenic Potential in Supraphysiological Administration: A Literature Review. J. Clin. Med..

[31] Coviello A.D., Kaplan B., Lakshman K.M., Chen T., Singh A.B., Bhasin S. (2008). Effects of Graded Doses of Testosterone on Erythropoiesis in Healthy Young and Older Men. J. Clin. Endocrinol. Metab..

[32] Cervi A., Balitsky A.K. (2017). Testosterone use causing erythrocytosis. Can. Med. Assoc. J..

[33] Basaria S., Coviello A.D., Travison T.G., Storer T.W., Farwell W.R., Jette A.M., Eder R., Tennstedt S., Ulloor J., Zhang A. (2010). Adverse Events Associated with Testosterone Administration. N. Engl. J. Med..

[34] Finkle W.D., Greenland S., Ridgeway G.K., Adams J.L., Frasco M.A., Cook M.B., Fraumeni J.F., Hoover R.N. (2014). Increased Risk of Non-Fatal Myocardial Infarction Following Testosterone Therapy Prescription in Men. PLoS ONE.

[35] Vigen R. (2013). Association of Testosterone Therapy with Mortality, Myocardial Infarction, and Stroke in Men with Low Testosterone Levels. JAMA.

